# Sinapic Acid Promotes Browning of 3T3-L1 Adipocytes via p38 MAPK/CREB Pathway

**DOI:** 10.1155/2020/5753623

**Published:** 2020-04-08

**Authors:** In-Seon Bae, Sang Hoon Kim

**Affiliations:** Department of Biology, College of Sciences, Kyung Hee University, Seoul 02447, Republic of Korea

## Abstract

Sinapic acid is a plant-derived phenolic compound, which acts as an antioxidant, anticancer, and anti-inflammatory agent. Although sinapic acid is valuable in a variety of therapeutic applications, its role in the improvement of obesity-related metabolic disease is relatively unexplored. Brown-like adipocytes (beige adipocytes) are characterized by a high concentration of mitochondria and high expression of uncoupling protein 1 (UCP1), which has specific functions in energy expenditure and thermogenesis. This study assessed the browning effects of sinapic acid in 3T3-L1 adipocytes. We investigated the expression of beige marker genes in 3T3-L1 adipocytes treated with sinapic acid. Sinapic acid increased the expression of peroxisome proliferator-activated receptor *γ* coactivator-1*α* (PGC-1*α*) and UCP1. Sinapic acid also promoted mitochondrial biogenesis by dose-dependently upregulating the oxygen consumption rate and enhancing the expression of representative subunits of oxidative phosphorylation complexes. In addition, treatment with p38 mitogen-activated protein kinase (MAPK) inhibitor and cAMP response element binding (CREB) inhibitor decreased the expressions of genes associated with thermogenesis, mitochondrial biogenesis, and oxidative phosphorylation. In summary, sinapic acid initiates browning 3T3-L1 adipocytes via the p38 MAPK/CREB signaling pathway. Thus, sinapic acid may have potential therapeutic implication in obesity.

## 1. Introduction

Obesity arising from overaccumulation of fat in the body is caused by the imbalance between energy absorption and release. In mammals, adipose tissue is functionally composed of two types of fat: white fat and brown fat [1]. Unlike white adipose cells, which store fat as an energy source, brown fat cells break down stored fat to release heat [2, 3]. Brown adipocytes express uncoupling protein 1 (UCP1), which promotes mitochondrial thermogenesis [3]. In addition to white and brown adipocytes, when the sympathetic nerves or *β*-adrenergic receptors are activated by exercise and cold or spicy foods, white adipocytes are converted into beige adipocytes through the expression of UCP1 and PGC-1*α* [4, 5]. Beige fat cells contain large numbers of mitochondria and small lipid droplets and oxidize fat to generate heat [6, 7].

Sinapic acid is a phenolic compound found in fruits, vegetables, and grains such as rice, oats, and buckwheat and is found in high concentrations in fermented foods such as wine and vinegar [8–11]. Phenolic compounds have beneficial effects on human health such as antioxidant and antimicrobial properties [11, 12]. Sinapic acid is known to have a high antioxidant activity [13, 14]. Treatment of human prostate cancer cells with sinapic acid reduces the expression of CDH2, MMP2, and MMP9, thus exhibiting anticancer effects [15]. In addition, sinapic acid plays a role in anti-inflammatory activities by inhibiting NF-*κ*B-mediated secretion of the cytokines IL-6 and IL-8 [16]. Sinapic acid is also known to have antibacterial effects against *Escherichia coli* and *Salmonella enterica* [17]. However, it is not yet known how sinapic acid affects obesity. Recently, several phenolic compounds have been reported to respond to *β*-adrenergic receptors [18]. Resveratrol, a phenolic compound, activates the *β*-adrenergic receptor, and oleuropein aglycone in raw olives activates *β*-adrenergic signaling, thereby increasing UCP1 expression in high-fat-diet-induced obese rats [19]. Therefore, in this study, we investigated whether sinapic acid is involved in the conversion of white adipocytes into beige adipocytes.

## 2. Materials and Methods

### 2.1. Cell Culture and Adipocyte Differentiation

3T3-L1 preadipocytes were cultured in Dulbecco's modified Eagle's medium (DMEM; Welgene, Daegu, Korea) supplemented with 10% bovine calf serum and 1% penicillin-streptomycin (Hyclone, Logan, UT, USA) in a humidified atmosphere containing 5% CO_2_ at 37°C. Cells were subcultured at 70% confluence. At two days postconfluence, cells were stimulated with DMEM containing 10% fetal bovine serum (FBS; Hyclone), 0.5 mM isobutylmethylxanthine (Sigma, Saint Louis, MO, USA), 1 *μ*M dexamethasone (Sigma), and 10 *μ*g/ml insulin (Sigma) (called d0) to induce differentiation. After 2 days, the medium was replaced with DMEM supplemented with FBS and insulin (10 *μ*g/ml) (called d2). Subsequently, the medium was replaced with DMEM containing 10% FBS without insulin every 2 days until day 6. Sinapic acid was added to the 3T3-L1 cells on d0 and d2. For p38 MAPK inhibition or CREB inhibition, 3T3-L1 adipocytes were incubated with 10 *μ*M of SB203580 (Sigma) or 10 *μ*M of 666-15 (Tocris Bioscience, Bristol, UK) for 24 h, respectively. After the treatment, the cells were harvested, and the total protein and RNA were extracted for subsequent analysis.

### 2.2. Cell Viability Assay

Cell viability was measured using the EZ-CyTox (Daeil Lab Service, Seoul, Republic of Korea). 3T3-L1 preadipocytes were seeded into 96-well plates at a density of 3 × 10^3^ cells per well. The cells were treated with sinapic acid (1 *μ*M to 20 *μ*M) or with dimethyl sulfoxide (DMSO), as a control and were incubated for 48 h. 3T3-L1 cells were added with a medium containing EZ-CyTox solution (0.01 ml/well) and incubated at 37°C for 1 h. Absorbance was measured at 450 nm using a Vmax microplate spectrophotometer (Molecular Devices, Sunnyvale, CA, USA).

### 2.3. RNA Isolation and Quantitative Real-Time Polymerase Chain Reaction

Total RNA was extracted from differentiated cells using TRIsure reagent (Bioline, London, UK) and was reverse-transcribed by M-MLV reverse transcriptase (Promega, Madison, WI, USA) and random primer. Quantitative real-time polymerase chain reaction (qRT-PCR) was performed with Roto gene Q PCR instrument (Qiagen, Hilden, Germany) using SensiFAST SYBR No-ROX Kit (Bioline). PCR conditions were as follows: 2 min at 95°C; 40 cycles of 4 s at 95°C, 10 s at 60°C, and 15 s 72°C. Primers were used as follows: UCP1 F: 5′-AGGCTTCCAGTACCATTAGGT-3′ and R: 5′-CTGAGTGAGGCAAAGCTGATTT-3′; PGC-1*α* F: 5′-TATGGAGTGACATAGAGTGTGCT-3′ and R: 5′-CCACTTCAATCCACCCAGAAA G-3′; CITED1 F: 5′-AACCTTGGAGTGAAGGATCGC-3′ and R: 5′-GTAGGAGAGCCTATTGGAGATGT-3′; HSPB7 F: 5′-GAGCATGTTTTCAGACGACTTTG-3′ and R: 5′-CCGAGGGTCTTGATGTTTCCTT-3′; TNFRSF9 F: 5′-CGTGCAGAACTCCTGTGATAA C-3′ and R: 5′-GTCCACCTATGCTGGAGAAGG-3′; EAR2 F: 5′-GAGGACGATTCGGCGTCA C-3′ and R: 5′-GTAATGCTTTCCACTGGACTTGT-3′; CD40 F: 5′-TGTCATCTGTGAAAAGGTGGTC-3′ and R: 5′-ACTGGAGCAGCGGTGTTATG-3′; NRF-1 F: 5′-TCTGTGCTTTCCAGCCACAA-3′ and R: 5′-TCCCACCCCTCCCTTATCAC-3′; TFAM F: 5′-ATTCCGAAGTGTTTTTCCAGCA-3′ and R: 5′-TCTGAAAGTTTTGCATCTGGGT-3′; NDUFB8 F: 5′-TGTTGCCGGGGTCATATCCTA-3′ and R: 5′-AGCATCGGGTAGTCGCCATA-3′; SHDB F: 5′- 5′-AATTTGCCATTTACCGATGGGA-3′ and R: 5′-AGCATCCAACACCATAGGTCC-3′; UQCRC2 F: 5′-AAAGTTGCCCCGAAGGTTAAA-3′ and R: 5′-GAGCATAGTTTTCCAGAGAAGCA-3′; COXIV F: 5′-ATT GGC AAG A GA GCC ATT TCT AC-3′ and R: 5′-CAC GCC GAT CAG CGT AAG T-3′; ATP5AF: 5′-TCT CCA TGC CTC TAA CAC TCG-3′ and R: 5′-CCA GGT CAA CAG ACG TGT CAG-3′; and *β*-actin F: 5′-GTGACGTTGACATCCGTAAAGA-3′ and R: 5′-GCCGGACTCATCGTACTCC-3′. Relative quantification of gene expression was normalized to *β*-actin expression. The relative expression was calculated using the 2^−ΔΔCt^ method.

### 2.4. Western Blot Analysis

Cell lysates were prepared in cell lysis buffer (Sigma) containing 50 mM sodium fluoride, 0.2 mM sodium orthovanadate, and 1% protease inhibitor. Total protein was separated by sodium dodecyl sulfate-polyacrylamide gel electrophoresis and transferred to a nitrocellulose membrane (Millipore, Bedford, MA, USA). After blocking with 5% skim milk at room temperature for 1 h, the membranes were incubated with primary antibodies against UCP1 (Abcam, Cambridge, MA, USA), PGC-1*α* (Boster Bio, Pleasanton, CA), TFAM (Cell Signaling, Beverly, MA), NDUFB8 (Invitrogen, Carlsbad, CA, USA), SDHB (Invitrogen), UQCRC2 (Abcam), COXIV (Abcam), ATP5A (Abcam), p38 MAPK (Cell Signaling), phospho-p38 MAPK (Cell Signaling), CREB (Abcam), phospho-CREB (Abcam), AKT (Abcam), phospho-AKT (Abcam), phospho-AMPK (Cell Signaling), and *β*-actin (Sigma) at 4°C overnight. The membranes were then incubated with horseradish peroxidase-conjugated anti-mouse IgG or anti-rabbit IgG antibodies at room temperature for 30 min. Membranes were developed with chemiluminescent HRP substrate (Advansta Inc, Menlo Park, CA, USA) using X-ray films.

### 2.5. Oxygen Consumption Rate (OCR) Assay

3T3-L1 cells were seeded in an XF24 microplate (Seahorse Bioscience, Billerica, MA, USA) at a density of 2,000 cells/well. After differentiation, cells were washed thrice with phosphate-buffered saline and incubated in XF base medium with 45 mM glucose, 4 mM glutamine, and 1 mM sodium pyruvate in a non-CO_2_ incubator for 1 h. The respiration was measured under basal conditions, followed by the sequential addition of 1 *μ*M oligomycin, 0.75 *μ*M carbonyl cyanide p-trifluoromethoxyphenylhydrazine, and 1 *μ*M rotenone/antimycin A. OCR measurements were performed using an XF analyzer (Seahorse Bioscience).

### 2.6. Statistical Analysis

Data were presented as mean ± standard deviation. The statistical analysis was performed using GraphPad Prism program. Student *t*-test or analysis of variance (ANOVA) was performed, and statistical significance was assessed at *P* values < 0.05 or *P* < 0.01.

## 3. Results

### 3.1. Sinapic Acid Upregulates the Expression of Beige Adipocyte-Related Genes

**Figure 1:**
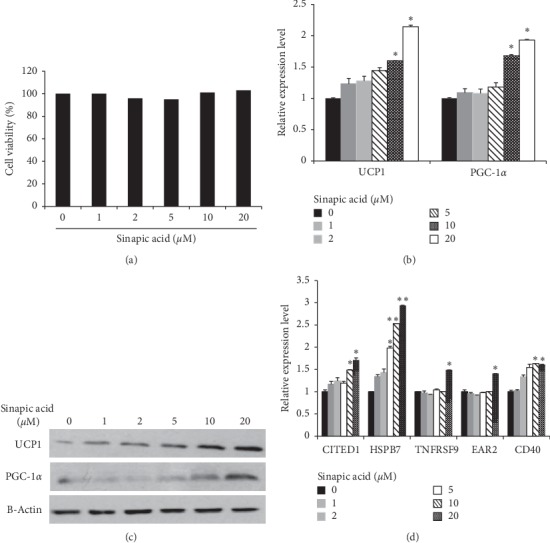
Browning of adipocytes in 3T3-L1 cells was induced by sinapic acid. (a) 3T3-L1 cells were treated with different concentrations of sinapic acid and cell viability was measured. (b, c) The expression of thermogenic genes (UCP1 and PGC-1*α*) was analyzed by qRT-PCR analysis (b) and western blot analysis (c) in 3T3-L1 adipocytes treated with sinapic acid. ^*∗*^*P* < 0.05. (d) The relative mRNA expression levels of beige-specific genes (CITED1, HSPB7, TNFRSF9, EAR2, and CD40) were measured by qRT-PCR in 3T3-L1 cells treated with different doses of sinapic acid. ^*∗*^*P* < 0.05, ^*∗∗*^*P* < 0.01.

First, we determined whether sinapic acid had an adverse effect on adipocyte viability. 3T3-L1 cells were treated with sinapic acid at concentrations of 1 to 20 *μ*M, and cell viability was measured. No difference in cell survival was seen, regardless of sinapic acid concentration (Figure 1(a)). Thus, the viability of 3T3-L1 cells was determined to not be affected by sinapic acid. Next, the effect of sinapic acid on adipocyte differentiation was investigated. The expression of PPAR*γ*, a master regulator of adipogenesis, increased in differentiation stages regardless of sinapic acid (Supplementary [Supplementary-material supplementary-material-1]). Lipolytic genes, HSL and ATGL, increased when the cells were treated with sinapic acid (Supplementary [Supplementary-material supplementary-material-1]). To investigate whether sinapic acid induces the transformation of white adipocytes to beige adipocytes, genes specifically expressed in beige adipocytes were examined. The mRNA levels of UCP1 and PGC-1*α*, thermogenic genes, were investigated in 3T3-L1 cells treated with sinapic acid. As shown in Figure 1(b), the expression of UCP1 and PGC-1*α* mRNA was significantly increased when cells were treated with sinapic acid at a concentration of 10 *μ*M or higher. UCP1 and PGC-1*α* protein levels were also increased in a sinapic acid-dependent manner (Figure 1(c)). The expression of CITED1, HSPB1, TNFRSF9, EAR2, and CD40 genes, all markers of beige adipocytes, was significantly increased in cells treated with 20 *μ*M of sinapic acid (Figure 1(d)). These results indicate that sinapic acid can induce browning of 3T3-L1 adipocytes by increasing the expression of beige adipocyte-related genes without cytotoxicity.

### 3.2. Sinapic Acid Induces Mitochondrial Biogenesis

**Figure 2:**
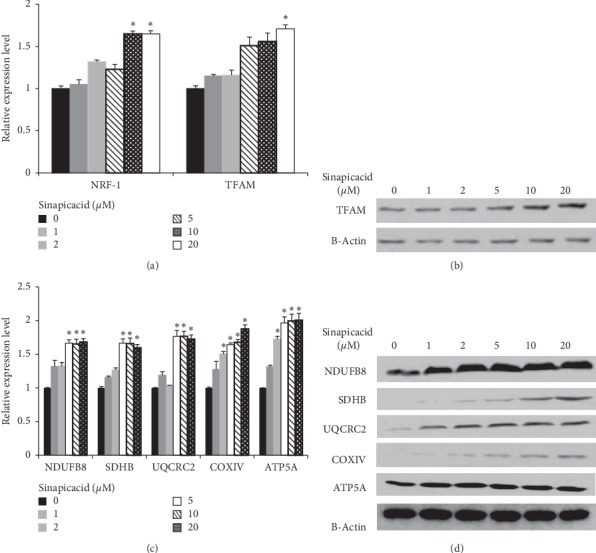
Sinapic acid stimulates mitochondrial biogenesis. (a) The effect of sinapic acid on NRF-1 and TFAM expression was analyzed in sinapic acid-treated 3T3-L1 adipocytes. ^*∗*^*P* < 0.05. (b) The protein level of TFAM in 3T3-L1 cells treated with sinapic acid was measured by western blot analysis. (c, d) The expression of mitochondrial biogenesis related genes (NDUFB8, SHDB, UQCRC2, COXIV, and ATA5A) in sinapic acid-treated 3T3-L1 adipocytes was detected by qRT-PCR analysis (c) and western blot analysis (d) ^*∗*^*P* < 0.05.

Induction of beige fat in white adipocytes is generally associated with mitochondrial biogenesis. We therefore investigated the effects of sinapic acid on mitochondrial biogenesis in 3T3-L1 cells. First, we examined the expression of TFAM and NRF-1 genes, the executors of mitochondrial biogenesis in 3T3-L1 cells. As shown in Figure 2(a), mRNA levels of NRF-1 and TFAM were significantly increased when cells were treated with 20 *μ*M of sinapic acid. Protein levels of TFAM increased with increasing concentrations of sinapic acid (Figure 2(b)). In order to observe the changes in oxidative phosphorylation (OXPHOX) which accompany mitochondrial biogenesis, we examined the expression of subunit genes of each complex in the mitochondrial electron transport chain. The mRNA expressions of NDUFB8 (Complex I), SDHB (Complex II), UQCRC2 (Complex III), COXIV (Complex IV), and ATP5A (Complex V) were increased following treatment with sinapic acid. This increase was especially noticeable in cells treated with 5 *μ*M or higher concentration of sinapic acid (Figure 2(c)). At the protein level, the expression of these genes increased as the concentration of sinapic acid increased in 3T3-L1 adipocytes (Figure 2(d)). These results suggest that sinapic acid promotes mitochondrial biogenesis in 3T3-L1 cells.

### 3.3. Sinapic Acid Increases Oxygen Consumption

**Figure 3:**
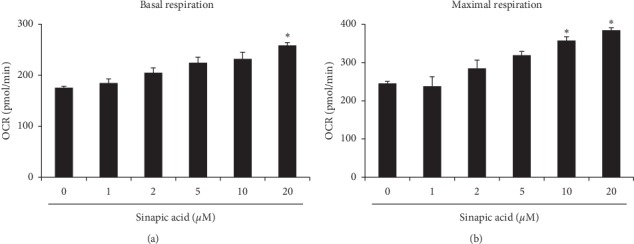
Sinapic acid promotes mitochondrial activity. Cells were cultured in different concentrations of sinapic acid. Mitochondrial respiration of 3T3-L1 adipocytes was measured using the XF-24 Extracellular Flux Analyzer. (a) Basal respiration was analyzed before the sequential addition of complex inhibitors in the mitochondrial electron transport chain. ^*∗*^*P* < 0.05. (b) Maximal respiration was assessed after the addition of carbonyl cyanide p-trifluoromethoxyphenylhydrazine as an uncoupling agent. ^*∗*^*P* < 0.05.

Because mitochondrial biogenesis was stimulated by treatment with sinapic acid, we measured the oxygen consumption rate to investigate the level of improvement in mitochondrial function caused by sinapic acid. This investigation showed a significant increase in basal respiration levels in cells treated with 20 *μ*M of sinapic acid, compared to the control group (Figure 3(a)). In addition, maximal oxygen consumption rate in 3T3-L1 cells was measured after the sequential injection of oligomycin and carbonyl cyanide p-trifluoromethoxyphenylhydrazine, a mitochondrial oxidative phosphorylation uncoupler. As shown in Figure 3(b)), maximal oxygen consumption rate was significantly increased in cells treated with 10 *μ*M or higher concentration of sinapic acid, compared to the control group. These results suggest that sinapic acid improves mitochondrial activity in 3T3-L1 cells.

### 3.4. Effect of Sinapic Acid on Mitochondrial Biogenesis Is Mediated by p38 MAPK/CREB Pathway

**Figure 4:**
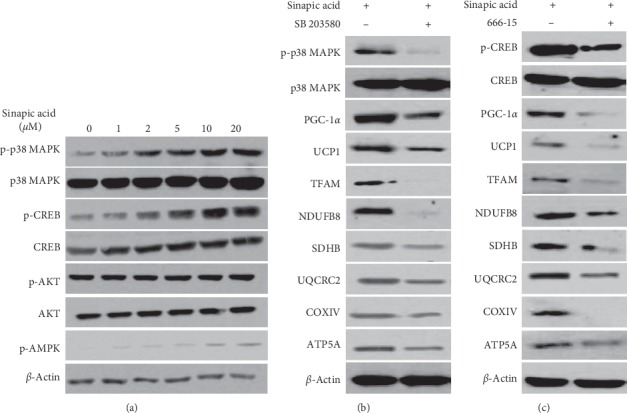
p38 MAPK and CREB mediate the browning effect of sinapic acid in 3T3-L1 cells. (a) 3T3-L1 adipocytes were treated with sinapic acid at the indicated concentrations. The levels of p38 MAPK, CREB, AKT, and AMPK proteins were measured by western blotting. (b, c) Cells exposed to 20 *μ*M sinapic acid were treated with the p38 MAPK inhibitor SB 203580 (b) and the CREB inhibitor 666-15 (c). The level of mitochondrial biogenesis-related genes and thermogenic genes in 3T3-L1 adipocytes were analyzed by western blot analysis.

We also investigated the pathway of mitochondrial biogenesis induced by sinapic acid during the conversion of white adipocytes into beige adipocytes. The phosphorylation of AMPK, p38 MAPK, CREB, and AKT proteins in 3T3-L1 cells was observed after treatment with sinapic acid. This showed that the levels of phosphorylated p38 MAPK protein and CREB protein increase as the concentration of sinapic acid increased. AMPK phosphorylation in cells treated with sinapic acid also increased slightly. However, AKT phosphorylation was not altered by sinapic acid (Figure 4(a)). In particular, the activation of p38 MAPK and CREB was significantly increased by sinapic acid. Next, we examined the expression of thermogenic genes and OXPHOX subunit genes by treating inhibitors in these pathways. Expression of both the thermogenic UCP1 and PGC-1*α* genes and the mitochondrial electron transport chain subunit genes, which were increased by sinapic acid, was decreased following treatment with the p38 MAPK inhibitor SB 203580 and the CREB inhibitor 666-15 (Figures 4(b) and 4(c)). Taken together, these results indicate that sinapic acid promotes mitochondrial biogenesis and mitochondrial activity through the p38 MAPK/CREB pathway, leading to the formation of beige adipocytes.

## 4. Discussion

Sinapic acid is a hydroxycinnamic acid synthesized from Mavolanate-Shikimate biosynthesis in plants. Hydroxycinnamic acids are composed of sinapic acid, coumaric acid, caffeic acid, and cinnamic acid and are known to inhibit adipogenesis in adipocytes [20]. In 3T3-L1 cells treated with coumaric acid, lipid accumulation is reduced and expression of lipoprotein-related genes is decreased [21]. Caffeic acid also inhibits the expression of adipogenesis-related genes such as aP2 and Fas in 3T3-L1 cells [22]. In relation to brown fat induction, it has been reported that treatment of 3T3-L1 adipocytes with trans-cinnamic acid increases UCP1 and PGC-1*α* expression leading to a brown-like phenotype [23]. This study showed that the expression of beige-specific genes also increased following treatment with sinapic acid. Together, these results indicate that several hydroxycinnamic acids such as sinapic acid, caffeic acid, and trans-cinnamic acid induce a brown-like phenotype in adipocytes.

Although the mechanism of action is not known precisely, sinapic acid is known to have the highest antioxidative effect among hydroxycinnamic acids [24, 25]. For example, 2,2′-azobis (2-amidinopropane) dihydrochloride, which induces hemolysis, is administered to human erythrocytes to measure the degree of cell damage by free radicals. Sinapic acid has the lowest IC50 value for hemolysis inhibition of all the hydroxycinnamic acids, including caffeic acid, coumaric acid, and ferulic acid [24]. In addition to antioxidant effects, sinapic acid showed higher ability for brown fat induction than other hydroxycinnamic acids. For example, 200 *μ*M of trans-cinnamic acid is required for the browning effect of 3T3-L1 white adipocyte [23], but in this study, the expression of beige fat-related genes was increased by treatment with 20 *μ*M of sinapic acid. Sinapic acid can induce a brown-like phenotype at concentrations ten times lower than trans-cinnamic acid. The fact that these plant-derived substances are effective even at low concentrations in cells has the advantage of reducing side effects in future clinical trials. Therefore, sinapic acid could be effectively used as a potential antiobesity agent.

Mitochondrial biogenesis is regulated by several pathways including AMPK, p-38 MAPK, CREB, and AKT [26–29]. PGC-1*α* is a major gene in mitochondrial biogenesis. AMPK, p38 MAPK, and CREB pathways increase PGC-1*α* expression, while AKT decreases PGC-1*α* expression [30, 31]. In this study, sinapic acid did not alter the AKT pathway, but activated AMPK, p38 MAPK, and CREB pathway. In other words, sinapic acid induces PGC-1*α* expression through various pathways, whereas trans-cinnamic acid in 3T3-L1 cells increases expression of PGC-1*α* through an AMPK pathway [23], suggesting that sinapic acid is more capable of brown fat induction than trans-cinnamic acid. However, it seems that sinapic acid does not act on the same pathway in all cells. In human hair follicle dermal papilla cells, sinapic acid induces AKT activation and promotes hair growth [32], whereas in rat chondrocytes, sinapic acid inhibits IL-1*β* induced MAPK, thereby reducing inflammation [33]. These data indicate that the mechanism of sinapic acid varies from cell to cell.

## 5. Conclusions

In conclusion, our results suggest that, in 3T3-L1 adipocytes, sinapic acid stimulates mitochondrial biogenesis via the AMPK, p38 MAPK, and CREB pathways, leading to browning of white adipocytes. Although further studies are required, sinapic acid can potentially be used in the effective treatment of obesity.

## Data Availability

The data used to support the findings of this study are available from the corresponding author upon request.

## Supplementary Materials

Supplementary Materials Supplementary Figure 1: PPAR*γ* expression is not altered by sinapic acid in 3T3-L1 cells. In the Materials and Methods section, a method for inducing adipocyte differentiation is presented. At two days postconfluence, cells were stimulated with differentiation medium (day 0). Samples were harvested every two days while inducing differentiation for six days. The expression level of PPAR*γ* mRNA was measured in adipocytes treated with sinapic acid. Supplementary Figure 2: the expression of lipolytic genes is increased in cells treated with sinapic acid. (a) 3T3-L1 cells were treated with different concentrations of sinapic acid. The relative mRNA expression levels of lipolytic genes (HSL and ATGL) were analyzed by qRT-PCR analysis. ^*∗*^*P* < 0.05, ^*∗∗*^*P* < 0.01.
