# Supplementary material for: Sinapic Acid Promotes Browning of 3T3-L1 Adipocytes via p38 MAPK/CREB Pathway
Source: Biomed Res Int. 2020 Apr 8;2020:5753623. doi: 10.1155/2020/5753623 (PMC7171644; doi:10.1155/2020/5753623)
Supplement: Supplementary Materials — Supplementary Figure 1: PPARγ expression is not altered by sinapic acid in 3T3-L1 cells. In the Materials and Methods section, a method for inducing adipocyte differentiation is presented. At two days postconfluence, cells were stimulated with differentiation medium (day 0). Samples were harvested every two days while inducing differentiation for six days. The expression level of PPARγ mRNA was measured in adipocytes treated with sinapic acid. Supplementary Figure 2: the expression of lipolytic genes is increased in cells treated with sinapic acid. (a) 3T3-L1 cells were treated with different concentrations of sinapic acid. The relative mRNA expression levels of lipolytic genes (HSL and ATGL) were analyzed by qRT-PCR analysis. ∗P < 0.05, ∗∗P < 0.01. [file 5753623.f1.docx]

Supplementary Figure 1.


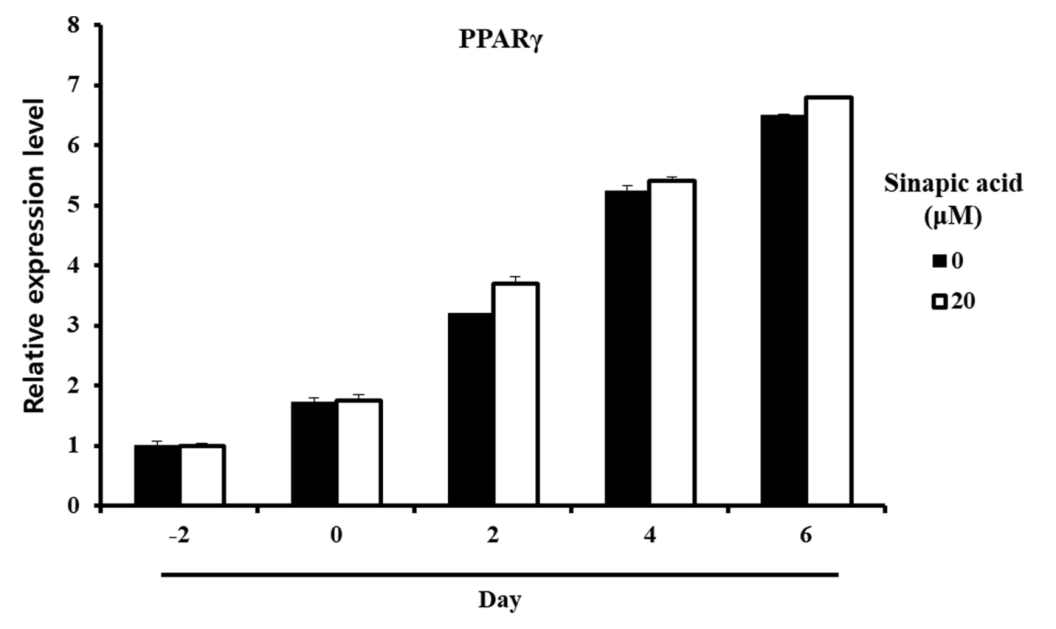


Supplementary Figure 1: PPARγ expression is not altered by sinapic acid in 3T3-L1 cells. In the materials and methods section, a method for inducing adipocyte differentiation is presented. At two days post-confluence, cells were stimulated with differentiation medium (day 0). Samples were harvested every two days while inducing differentiation for six days. The expression level of PPARγ mRNA was measured in adipocytes treated with sinapic acid.

Supplementary Figure 2.


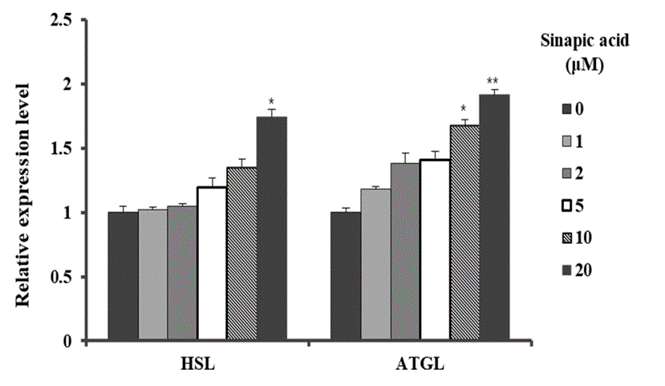


Supplementary Figure 2: The expression of lipolytic genes is increased in cells treated with sinapic acid. (a) 3T3-L1 cells were treated with different concentration of sinapic acid. The relative mRNA expression levels of lipolytic genes (HSL and ATGL) were analyzed by qRT-PCR analysis. *P < 0.05, **P < 0.01.
